# The Lay Press and Surgery

**Published:** 1898-03

**Authors:** 


### Abstracts and Selections.

### The Lay Press and Surgery.

The question of the Old Testament, "Why do the people imagine a vain thing?" may be answered, at least in regard to medical matters, by reference to the lay press which, undoubtedly with the best motives, publishes as news everything from osteopathy and operations, which the uninformed reporter chronicles as absolutely unique, up to the much-vaunted extirpation of the stomach, enthusiastic reports of which have been scattered by special correspondents and the press agencies broadcast over the country.

Dr. Schlatter's report of the operation in the *Correspondenz-blatt fuer Schweizer Aerzte*, is very complete and most modest. He states that in his case the total extirpation of the stomach was only on account of the length and great elasticity of the esophageal end of the stomach. The patient was a woman, fifty-six years of age, a silk weaver by occupation, who had suffered from pain in the stomach from childhood, and had a family history of cancer of the stomach. The clinical history and physical examination pointed unmistakably to cancer of the stomach.

The tumor which could be easily felt, seemed to involve the entire stomach. Schlatter operated on September 6, 1897, under morphine-ether anesthesia, in the following manner: Incision in the median line from the ensiform cartilage to the umbilicus. The stomach immediately presented in the wound. The entire organ was diseased and converted into a hard mass; it was not adherent. The stomach was first freed from all its attachments at the greater and lesser curvatures; the omentum excised between Pean forceps, and silk sutures applied. The stomach was then forcibly dragged downward, a Woelfler clamp applied as high up as possible, and a Stille forceps secured to the cardiac end of the stomach. The stomach was then severed directly below the esophageal extremity. The same steps were repeated at the pyloric extremity of the stomach. The duodenum was next moved as far as possible towards the pancreas. After compression of the duodenum with tumor clamps, the entire stomach was removed between the two points of compression. A few lymphatic nodes were also dissected out of the greater curvature of the stomach. The patent lumen of the duodenum was protected by iodoform gauze. He next tried to pull the duodenal opening upward toward the esophageal cleft. It was only with considerable difficulty that the two could be made to touch. They could not be joined by direct suture; therefore the duodenal rim was invaginated and closed by a double suture. A coil of the intestine about fifteen inches below the severed end was then sutured to the end of the esophagus. A longitudinal incision about one inch in length was made in the bowel and the intestinal mucosa united to the mucous membrane of the esophagus by a continuous circular suture. Above this an outer circular suture was placed through the muscularis and serosa. The union was completed by a Lembert suture. The clamps were then removed, and the abdominal wound closed in the usual manner with silk. The operation lasted nearly two hours and a half. The specimen proved on examination to be a small-celled alveolar glandular carcinama.

The patient made an uninterrupted recovery, and at the time of the report was apparently in good health, and was working daily in the hospital.

Dr. Schlatter prefaces his report with a brief statement of the work previously done in this direction. Kroenlein excised at 22 cm. of the greater and 13 cm. of the lesser curvature of the stomach, with recovery. Maydi removed more than one-half, and Von Hacker more than two-thirds of the stomach. In the more recent so-called total extirpations, small portions—one or two square inches of the cardiac extremity—have invariably been left. In a case reported by Ewald in 1881 as total extirpation, the author was informed by Lindner that 2 cm. remained. The woman died of hemorrhage on the third day. In 1884 Langenbuch made a probably total resection of the stomach, but the woman died on the sixth day. In another case he removed seven-eights of the stomach, the remainder, consisting of the cuff-like esophageal and cardiac ends, being united by sutures; this patient recovered. Schuhardt removed all but "three fingers" of the cardiac portion, and the patient enjoyed perfect health for two and one-half years and then died. At the autopsy the stomach was found to have enlarged to a capacity of 500 cc.

Experiments on animals have been performed by many operators. Czerny, in 1876, performed gastrectomy on five dogs, four of which promptly died; the fifth was fed at first on small portions of milk and pulverized meat, and later, after two months, was put on a full diet. The animal's weight at the time of operation was 5,850 grams; a month later, 4,490 grams, and ten months later 7,000 grams. The physiological functions were perfectly normal. Six years later the dog was killed, and a very small piece of the cardiac end of the stomach found remaining, which formed a spherical organ filled with food. Pechon and Carvello reported in 1893 a gastrectomy on a dog performed five months before. The dog ate slowly, but his diet was not limited. Since the operation he had gained 500 grams. In 1896 Monari reported a total extirpation of the stomach in a dog. The operation was followed by occasional vomiting, which finally ceased. The animal was put on regular canine diet and had decreased slightly in weight. Filippi made a similar report in 1896. The dog was killed a year later. Autopsy showed enlargement of the lower portion of the esophagus and the upper portion of the duodenum, and in the upper two-thirds of the duodenum tbe microscope revealed thickening of the annular muscular fibers.

From Dr. Schlatter's article Dr. C. C. Wendt (*Medical Record*) draws the following conclusions:

1. The human stomach is not a vital organ.
2. The digestive capacity of the human stomach has been considerably overrated.
3. The fluids and solids constituting an ordinary mixed diet are capable of complete digestion and assimilation without the aid of the human stomach.

4. A gain in the weight of the human body may take place in spite of the total absence of gastric activity.

5. Typical vomiting may occur without a stomach.

6. The general health of a person need not immediately deteriorate on account of the removal of the stomach.

7. The most important office of the human stomach is to act as reservoir for the reception, preliminary preparation and propulsion of food and fluids. It also fulfils a useful purpose in regulating the temperature of swallowed solids and liquids.

8. The chemical function of the human stomach may be completely and satisfactorily performed by the other divisions of the alimentary canal.

9. Gastric juice is hostile to the development of many micro-organisms.

10. The free acid of normal gastric secretion has no power to arrest putrefactive changes in the intestinal tract. Its antiseptic and bactericide potency has been overestimated.

In striking contrast to the modest statements of Schlatter are the lay comments on his article. One prominent daily paper in Chicago stated in effect that Chicago would soon show the old world that American surgeons could do at least as much as the European operators in this department of surgery, the implication being apparently that in this country we would not only extirpate the stomach, but the entire digestive tract.

Since the matter has become public property, at least two American surgeons have performed the operation, which has been duly exploited by the lay press. In both cases the operation is said to have been a brilliant success, but unfortunately both patients died. "Many surgeons," says the report, "were present at the operations, and they all declared that the women would have lived had they been a little stronger."—*Medical Standard.*

---

**Boils in the External Auditory Canal.**—For the relief of pain, Field (*British Medical Journal*) believes that nothing is better than glycerine. It acts by relieving tension, and, when used, should be mixed with an equal quantity of tincture of opium, and some boric acid, applied on a small dossil of cotton.

